# Mapping the spatial disparities of HIV prevalence in Ethiopian zones using the generalized additive model

**DOI:** 10.1038/s41598-024-55850-8

**Published:** 2024-03-14

**Authors:** Seyifemickael Amare Yilema, Yegnanew A. Shiferaw, Alebachew Taye Belay, Denekew Bitew Belay

**Affiliations:** 1 Department of Statistics, College of Natural and Computational Science, Debre Tabor University, P.O. Box 272, Debre Tabor, Ethiopia; 2 Department of Statistics, University of Johannesburg, Auckland Park Kingsway Campus, P.O. Box 524, Johannesburg, 2006 South Africa; 3 Department of Statistics, College of Science, Bahir Dar University, Bahir Dar, Ethiopia

**Keywords:** Generalized additive model, Spatial heterogeneity, HIV, Odds ratio, Zones, Diseases, Health care, Health occupations

## Abstract

HIV is a worldwide social and health pandemic that poses a significant problem. This study contributes to the 2030 global agenda of reducing HIV prevalence. The study analyzed HIV prevalence using the 2016 Ethiopian Demographic and Health Survey data. The study included men aged 15–54 years and women aged 15–49 years who responded to questions about HIV tests. A generalized geo-additive model (GAM) was fitted to HIV data using nonparametric smooth terms for geolocations. Two smoothing techniques were used in GAMs to evaluate spatial disparities and the probable effects of variables on HIV risk. There were certain areas in Ethiopia that were identified as hot spot zones for HIV, including Nuer and Agnuak in Gambella, West Wollega and Illubabor in Oromia, Benchi Maji and Shaka in SNNPR, Awsi, Fantana, Kilbet, and Gabi in the Afar region, Shinilie of the Somalia region, North and South Wollo, Oromia special zones of the Amhara region, Central Ethiopia, and Addis Ababa city. On the other hand, the eastern parts of Ethiopia, particularly most zones in the Somalia region, were identified as cold spot zones with the lowest HIV odds ratio. The odds of HIV+ were higher for those who reside in rural areas than in urban areas. Furthermore, people who have STIs, who used contraceptive methods, and who learned at the secondary level of education were more likely to be infected with HIV. After adjusting for confounding variables, the results indicated that there are substantially significant spatial variations in HIV prevalence across Ethiopian zones. These results provide essential information to strategically target geographic areas to allocate resources and policy interventions at zonal level administrations.

## Introduction

The human immune deficiency virus (HIV), which causes acquired immune deficiency syndrome (AIDS), is among the world's most critical public health threats^1^. There is an international commitment to stop new HIV infections and to ensure that everyone with HIV has access to treatment^1^. Since the beginning of the epidemic, 84.2 million people have acquired HIV and approximately 40.1 million people have died from the disease. There were 38.4 million HIV-positive people on the globe as of the end of 2021, among whom 36.7 million adults and 1.7 million children under the age of 15 are HIV-positive, 25.6 million of whom are in the African Region. In the world, 0.7% of adults between the ages of 15 and 49 are estimated to have HIV, yet the intensity of the epidemic continues to vary widely between various countries and regions. Based on WHO reports, 3.4% of the population in the African region, or more than two-thirds of all HIV-positive people worldwide, are still infected with the virus^2^.

Although only 11% of the world's population lives in sub-Saharan Africa, this region is the epicenter of the HIV/AIDS epidemic. A pattern investigation of the HIV/AIDS prevalence in Ethiopia between 1982 and 2000 revealed a consistent increase followed by a decline after 2000^3^. According to reports from the Ethiopian DHS, the prevalence of HIV among adults was 0.2% in 1985, 3.2% in 1995, 3.3% in 2000, 1.4% in 2005, 1.5% in 2011, and 0.9% in 2016^3–7^. The HIV prevalence was unevenly distributed among Ethiopia's regions, with Gambella, Addis Ababa, Central Oromia, Dire Dawa, Harari, and Afar being the most seriously affected regions^7–9^.

HIV/AIDS quickly became a pandemic after the first AIDS case was reported in Ethiopia in 1986^10^. Ethiopia, one of the most severely affected nations, has made significant investments in HIV/AIDS prevention and care since the 1990s. Ethiopia adopted the global target, which was originally introduced in 2014 by the Joint United Nations Programme on HIV/AIDS^11^, as one of the tactics intended to end the AIDS and HIV epidemics by 2030. Ethiopia has committed to lowering HIV infections and is working hard on a number of projects, including a national roadmap for HIV prevention and control in 2018^12^ and a national strategic plan for HIV/AIDS from 2021 to 2025 with the goal of having AIDS free Ethiopia by 2030^13^. This study can support the global 2030 agenda of ending HIV prevalence.

It is useful to map the crude and adjusted covariate spatial disparities of potential risk factors in spatial health studies for identifying infectious disease problems^14^. Complex spatial patterns associated with disease risks are exposed to substantial fluctuation due to sparsity. Smoothing provides an effective way to address these problems by reducing variability while allowing non-parametric estimates by borrowing strength from nearby observations. For mapping individual level epidemiological data, generalized additive models (GAMs), first introduced by Hastie and Tibshiran^15^, are frequently used model-based techniques^14,16–19^. When examining geographic variability in a flexible approach, GAMs offer a unified statistical framework that enables individual-level risk factor adjustment^14^. Modelling complicated spatial relationships is ideal for GAMs due to their versatility and intuitive smoothing approaches.

The Federal Ministry of Health decentralized the health service in accordance with the governmental structures (regions, zones, and woredas)^20,21^. Zones are the third level of governmental administrative hierarchies, responsible for operational planning, resource allocation, and healthcare implementation. Zonal governments act as a "milestone" between woreda (district) and regional administrations. Additionally, the zonal health department is responsible for monitoring and evaluating health activities at the district level^22^. Therefore, analysis of HIV prevalence at the zonal level is a significant benefit for policy interventions.

HIV prevalence maps generated highlight the spatial disparities in the epidemic within sub-Saharan African (SSA) countries, and localized areas where both the burden and drivers of the HIV epidemic are concentrated^23^. Research findings suggested a large geographical variation in the HIV epidemic across the SSA countries^23–25^. The distribution of HIV infection in Ethiopia is varied in all its regions^7^. These studies are based on HIV prevalence disparities at national and regional levels. However, adjusted covariates are not often considered in studies of spatial disparities at the lower level of government structures. Based on works of literature, there are very limited studies on the geospatial HIV prevalence using the GAM model in Ethiopian zones. As a result, our focus is on mapping the spatial variations in HIV prevalence across different Ethiopian zones and investigating the effects of sociodemographic, biological, and behavioral covariates.

## Methods

### Data sources

The 2016 Ethiopian demographic and health survey data was used in this study. For the purpose of analyzing the HIV prevalence, the individual women’s and men’s records and HIV files were merged. Overall, household respondents (women aged 15–49 years and men aged 15–59 years) were used for the analysis. There are 21 sampling strata in the 2016 Ethiopian Demographic and Health Survey (EDHS). In two stages, samples of enumeration areas (EAs) were randomly chosen from each stratum. By classifying the sampling frame within each sampling stratum prior to sample selection, in accordance with administrative units at various levels, and by using a probability proportional to size selection at the first stage of sampling, implicit stratification and proportional allocation were achieved at each of the lower administrative levels^6^. Then after, 645 EAs were separately selected in each stratum during the first stage, with the likelihood being inversely correlated to the size of the EAs. Of the total 645 EAs, 202 EAs and 443 EAs were urban and rural, respectively. Finally, 28 households per cluster were chosen in the second stage using a systematic sampling method with equal probability from the newly created households.

In order to get capillary blood for HIV testing, interviewers pricked the fingers of household respondents. The anonymously linked protocol developed for the DHS program served as the foundation for the protocol for collecting and analyzing blood samples. In accordance with this protocol, the data from the HIV testing can be combined with the anonymized survey data file via a unique identifier to enable analyses of HIV status by sociodemographic characteristics. However, the results are anonymous and not given to the respondents^26^.

### Spatial data

**Figure 1:**
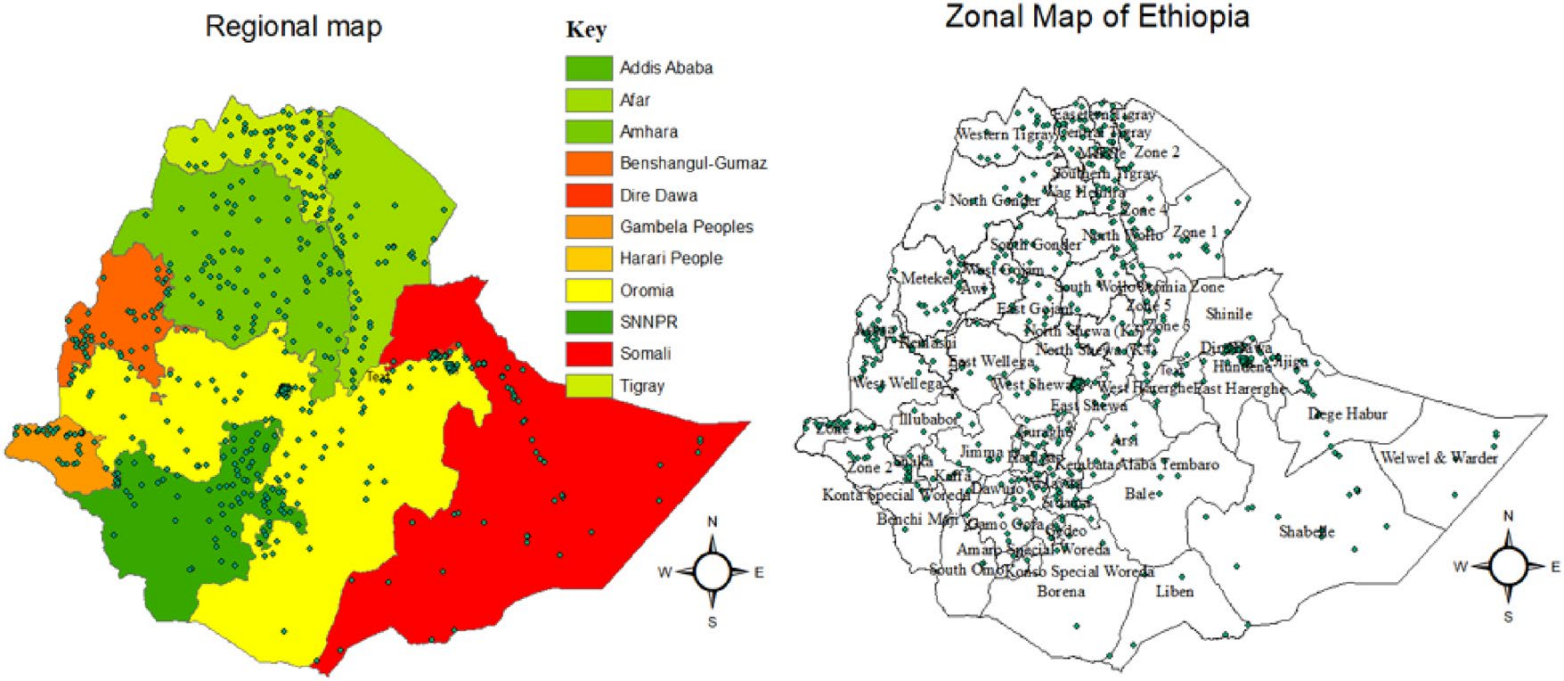
Regional and zonal maps of Ethiopia.

Ethiopia has been divided into nine regions and two administrative cities and further subdivided into 83 zones for administrative purposes. Each residential cluster housing was connected to all household attributes using GPS point data. For concerns of privacy of the respondents, the GPS urban/rural locations have been masked^27^. To protect the privacy of respondents living in small administrative units, clusters of urban residences were displaced by up to 2 km, while clusters of rural residences were displaced by up to 5 km. Additionally, 1% of rural clusters were displaced by up to 10 km^27^. Figure 1 shows the regional and zonal maps of Ethiopia; the dot-like points in the zonal maps were the EAs.

### Generalized geo additive statistical model

Generalized additive models (GAMs) were initially developed by Hastie and Tibshiran^15^. These models presuppose that a link function connects an additive predictor to the mean of the response variables. Similarly to the generalized linear model, generalized additive models allow any member of the exponential family of distributions for the response probability distribution. The GAM allows for an unknown smooth function in the linear predictor, the only distinction between GLM and GAM^15,28^. In general, GAM has the interpretability advantage over the general linear models (GLM) since it is substantially more flexible because the independent and dependent variables are not rigidly defined as linear^28–30^. The random component of the GLM identifies the probability distribution of response Y, which is assumed to belong to the exponential family with the density function of the form as follows.

$$p(y;\theta ;\phi ) = exp\left\{ {\frac{{[y\theta - b(\theta_{i} )]}}{a(\phi )} + c(y,\phi )} \right\}$$

where $\theta$ is the natural parameters of the exponential family, $\phi$ is a scale or dispersion parameter common to all respondents, $c\left( . \right)$ are functions depending on the specific exponential family. Let us define the respondents of HIV infectious for in *i*th EA as

$$y_{ij} = \left\{ {\begin{array}{*{20}l} {1,} \hfill & { if\quad \,respondents \,in \,the \,i{th} \,EA \,is \,HIV + } \hfill \\ {0,} \hfill & {Otherwise} \hfill \\ \end{array} } \right.$$

We consider modeling of respondents that are distributed on a map with spatial coordinates $u_{i}$ and $v_{i}$ denoting the geo location for the *i*th EAs for Ethiopian zones. Furthermore, the distribution of the outcome variable belongs to the exponential family, and therefore the GAM for a spatial effect analysis can be specified as

$$logit(\pi_{ij} ) = \eta_{ij} = X_{ij}^{t} \beta + S(u_{i} ,v_{i} ),i = 1,2, \ldots ,n$$

where $logit(\pi_{ij} )$ is the logit link function and Y is the response variables^31^. The response variable Y is the binary in HIV prevalence data and therefore, $logit(\pi_{ij} )$ is the logit link function. $\eta_{ij}$ is an additive predictor model, $\beta$ denotes a vector of coefficients associated with adjustment covariate $X_{ij}$. $S(u_{i} ,v_{j} )$ is a 2-dimentional non parametric smooth function that is used to model geographical location of respondents, a nonlinear function of geo location and finally $u_{i}$ and $v_{j}$ are the coordinates of longitude and latitude respectively^32^.

In the context of HIV data, GAMs have been used to evaluate spatial disparities and understand how sociodemographic, biological, and behavioral factors contribute to the spatial patterns of HIV prevalence^33^. This analysis primarily assesses the relationship between geographic location and HIV prevalence in Ethiopian zones. To achieve this, a 2-dimensional locally weighted regression smoother (LOESS) was used to smooth over the longitude and latitude of the respondent's geolocation^14,16^. The implementation of GAMs in this context involves several steps, including:

#### Primary data source

As stated in the data section, this study uses data from the 2016 EDHS. The data includes HIV test results and responses from men aged 15–59 and women aged 15–49 who participated as household respondents (Supplementary material).

#### Model fitting

As stated in the “Methods” section, GAMs were utilized to analyze the data in this study. GAMs incorporate nonparametric bivariate smooth terms of spatial location parameters, which are X and Y coordinates, to investigate the patterns without relying on strong assumptions about the underlying functions. The MapGAM package is used to fit a GAM and map smoothed spatial effect estimates from respondents to HIV risk. MapGAM was developed to provide a single R package that allows for estimating, predicting, and visualizing covariate-adjusted spatial effects using individual-level data. The package estimates covariate-adjusted spatial associations and the odds ratio of HIV prevalence via GAMs that include a non-parametric bivariate smooth term of geolocation parameters^14,30,34^.

#### Model evaluation

The least absolute shrinkage and selection operator (LASSO) regression technique was used to select significant variables for further analysis. It is commonly used for linear and logistic regression. The performance of the GAMs was evaluated using cross-validation, which helps to choose the best model based on AIC and deviance values.

#### Interpretation

The GAMs results can be used to identify high-risk HIV regions and analyze the impact of various factors on HIV prevalence^33^.

The GAM framework is a versatile and sturdy statistical method that can effectively analyze complex relationships between various factors and a dependent variable. This approach has proved to be helpful in studying HIV data as it can identify spatial disparities and potential factors contributing to the spatial patterns of HIV prevalence. The insights obtained from such analysis can help in designing intervention and control programs that are geographically targeted and more effective^33^.

## Results

The descriptive analysis of HIV coverage with the corresponding sociodemographic factors (such as region, place of residence, educational level, literacy, wealth index, and current marital status), biological factors (such as age at first sex and having a genital sore or ulcer in the last 12 months), and behavioral factors (such as ever using a contraceptive method and ever hearing of AIDS) of participants were presented in Table 1 below. The variables were chosen based on the review of works of literature^28,35–37^. Based on this study, Gambella (4.4%), Addis Ababa (3.48%), Dire Dawa (2.72%) and Harari (2.67%) were reported as having high percentages of HIV positives. On the other hand, Somalia (0.12%) and Southern nation’s nationalities and people’s region (SNNPR) (0.35%) reported low coverage of HIV negative in the country.

**Table 1:** Percentage distributions in social, biological and behavioral characteristics of HIV positive and HIV negative participants from EDHS data.

Variables	Categories	Frequency		Percentage	
		HIV−	HIV+	HIV− (%)	HIV+ (%)
Region	Tigray	2941	32	98.924	1.076
	Afar	1766	23	98.714	1.286
	Amhara	3479	42	98.807	1.193
	Oromia	3331	23	99.314	0.686
	Somalia	1664	2	99.880	0.120
	Benshangul	1965	18	99.092	0.908
	SNNPR	3333	12	99.641	0.359
	Gambella	1740	80	95.604	4.396
	Harari	1238	34	97.327	2.673
	Addis Ababa	2605	94	96.517	3.483
	Dire Dawa	1608	45	97.278	2.722
Place of residence	Urban	7891	288	96.479	3.521
	Rural	17,779	117	99.346	0.654
Educational level	No education	10,972	75	99.321	0.679
	Primary	9287	166	98.244	1.756
	Secondary	3698	119	96.882	3.118
	Higher	1713	45	97.440	2.560
Literacy	Cannot read at all	13,428	111	99.180	0.820
	Able to read	12,242	294	97.655	2.345
Wealth index	Poorest	6152	40	99.354	0.646
	Poorer	3750	26	99.311	0.689
	Middle	3427	25	99.276	0.724
	Rich	3637	26	99.290	0.710
	Richest	8704	288	96.797	3.203
Current marital status	Single	7334	132	98.232	1.768
	Married	15,888	203	98.738	1.262
	Other	2448	70	97.220	2.780
Ever heard of AIDS	No	1889	20	98.952	1.048
	Yes	23,781	385	98.407	1.593
Have you had a sexually transmitted infection (STI) in the last 12 month	No	25,587	398	98.468	1.532
	Yes	83	7	92.222	7.778
Had a genital sore/ulcer in last 12 months	No	25,157	394	98.458	1.542
	Yes	402	7	98.289	1.711
Ever been tested for HIV	No	13,178	122	99.083	0.917
	Yes	12,492	283	97.785	2.215
Age at first sex	Not had sex	6441	103	98.426	1.574
	Below 15	4053	59	98.565	1.435
	15–19	11,746	189	98.416	1.584
	Above 20	3430	54	98.450	1.550
Ever used contraceptive method	No	20,055	285	98.599	1.401
	Yes	5615	120	97.908	2.092
Number of sexual partners, excluding spouse, in last 12 months	Not had	24,901	370	98.536	1.464
	One sex partner	721	33	95.623	4.377
	Two and more	48	2	96.000	4.000
Number of sex partners, including spouse, in last 12 months	Not had	9348	176	98.152	1.848
	One sex partner	16,228	225	98.632	1.368
	Two and more	94	4	95.918	4.082

### Variable selection

Significant variables for further analysis were selected using least absolute shrinkage and selection operator (LASSO) regression. Nowadays LASSO is a widely used technique of variable selection for linear and logistic regression^38^. Consequently, region, place of residence, current marital status, educational level, had a sexually transmitted disease for the last 12 months, age at first sex, ever been tested for HIV/AIDS, ever heard HIV and ever used contraceptive method are selected as important variables for fitting GAM model. *PROC HPGENSELECT* in the SAS university edition is used for the selection process.

### Interpretations for geo spatial analysis

After controlling for disease related characteristics, the final GAMs result shows that individuals who lived in rural areas were 4 (AOR = 4.183, CI = 3.47, 4.90) times more likely being infected by HIV as compared to those living in urban areas (Table 2). Respondents who learned secondary education were 2 (AOR = 2.15, CI = 1.33, 2.97) times more likely to be infected by HIV than those who were none educated persons. Respondents who had any STI in the last 12 months prior to the survey were found to have higher odds of HIV+ (AOR = 4.34; CI = 3.09, 5.60) than those of STI-negative individuals. Individuals who were of other marital status (divorced, widowed, etc.) had higher odds of HIV+ than the single individuals. There were significant positive effects of contraceptive methods on HIV prevalence; the study found that those contraceptive users were (AOR = 1.23, CI = 1.04, 1.93) more likely to be HIV+ than those noncontraceptive users (Table 2).

**Table 2:** Adjusted HIV+ odds ratio and a 95% confidence interval for GAMs model.

		AOR	Lower	Upper
Place of residence	Urban (ref)			
	Rural	4.183	3.469	4.897
Educational level	No education (ref)			
	Primary	1.703	0.937	2.470
	Secondary	2.148	1.330	2.966
	Higher	1.471	0.554	2.388
Current marital status	Single			
	Married	0.840	− 0.062	1.742
	Other	1.294	1.052	2.237
Had any STI in the last 12 month	No (ref)			
	Yes	4.342	3.085	5.599
Age at first sex	Not had sex			
	Below 15	1.331	0.342	2.320
	15–19	1.238	1.12	2.164
	Above 20	0.844	− 0.129	1.817
Ever been tested for HIV	No (ref)			
	Yes	1.360	0.648	2.071
Ever heard of AIDS	No (ref)			
	Yes	0.547	− 0.443	1.537
Ever used contraceptive method	No (ref)			
	Yes	1.233	1.035	1.931

The *p* value (0.000) *used to test* the global spatial effect with the smallest deviance statistic shows that it is highly significant for the crude analysis, which indicates that the prevalence of HIV was significantly associated with the spatial locations (longitude, latitude) of the respondents. Before adjustment of covariates for spatial confounding, geographic variations were statistically significant. The optimal scan size, which minimized the AIC, was found to be 0.15, indicating that 15% of the adjacent dataset was used for smoothing the geo-location parameters (Table 3).

**Table 3:** Comparisons of unadjusted and adjusted covariate spatial effects with geo location coordinates.

Parameters	Unadjusted (crude)	Adjusted for covariates
Deviance statistic	3952.13	3795.30
AIC	3895.86	3751.33
*p* value	0.000	0.000
Span size	0.15	0.50
All variables effect predictions		
Minimum	− 7.84	− 3.07
1st quartile	− 1.23	− 0.87
Mean	− 0.74	− 0.29
3rd quartile	0.38	0.37
Maximum	2.00	1.46

**Figure 2:**
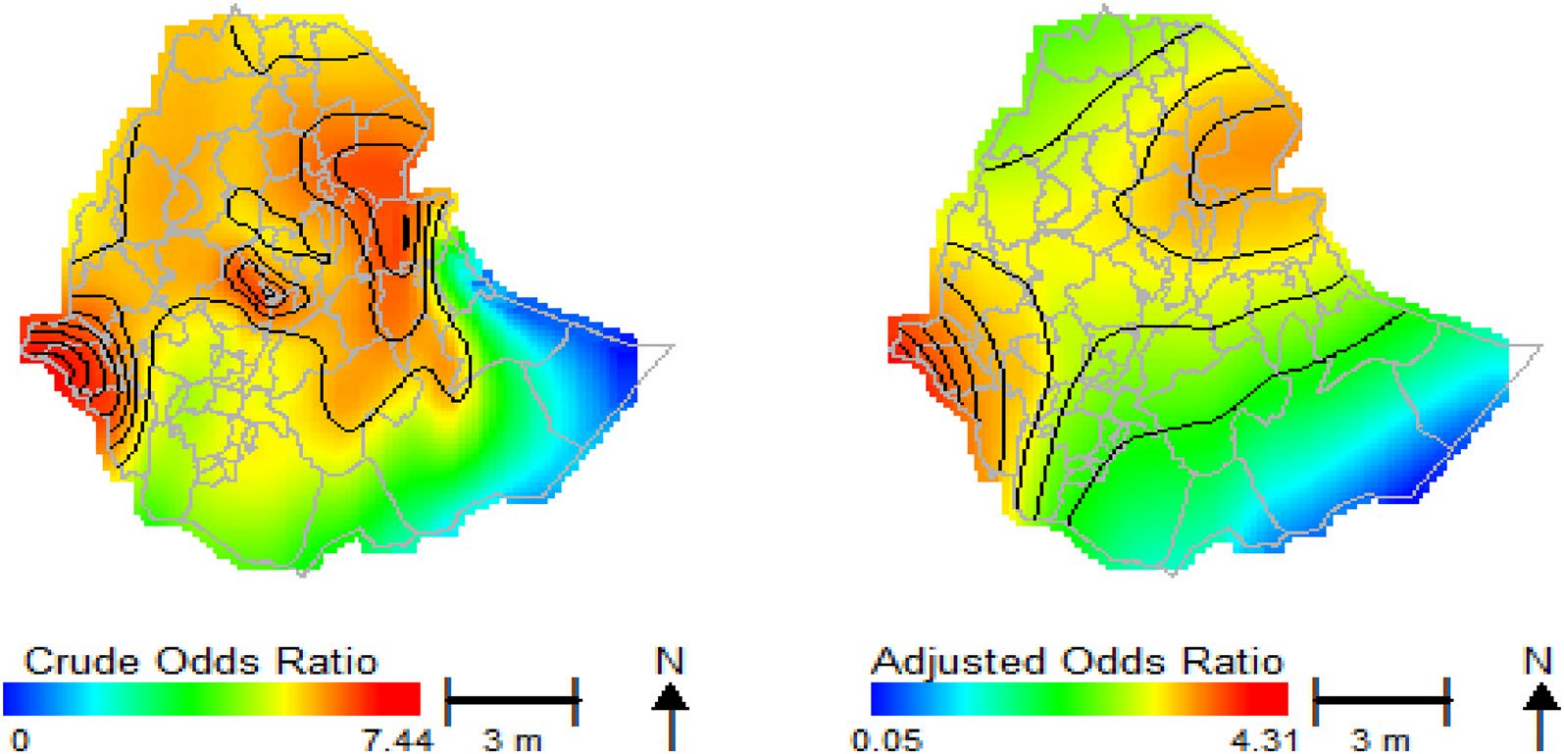
Map of the spatial distributions of crude HIV odds ratio (on the left) and adjusted covariates HIV odds ratio (at the right).

This study mapped both unadjusted (crude) and adjusted with covariate spatial confounding effects to explain the characteristics of HIV infectious to geo locations across Ethiopian zones (Fig. 2 at the left). The spatial distributions of the crude odds ratio (COR) map of HIV infectious shows the risk of HIV infections in different parts of the country. In western parts of the country, the hot spot zones were Nuer and Agnuak from the Gambella region, parts of the west Wollega and Illubabor of the Oromia region, parts of the Benchi Maji and Shaka of SNNPR, with the highest odds ratio. In the North Eastern parts of the country, Awsi, Fantana, Kilbet, parts of Hari and Gabi zones of Afar region, Shinilie of Somalia region, North and South Wollo, Oromia special zones of Amhara region have among the highest HIV odds ratio. Furthermore, in Central parts of Ethiopia, Addis Ababa city, West, East and North Shewa zones of the Oromia region have the highest HIV odds ratio (Maximum AOR is 7.44, global *p* value = 0.000 and span size = 0.15). In contrast, the cold spot zones were mostly found in the eastern parts of Ethiopia, particularly most zones in the Somalia region, which have the lowest HIV odds ratio.

The local test noticed areas with statistically and significantly higher and lower HIV prevalence, which are denoted by black lines in Fig. 2. The crude map showed a spatial pattern of HIV prevalence that was consistent with the geographic distributions of the respondents who had HIV+ results.

The spatial distribution of the covariate adjusted HIV odds ratio for respondents across Ethiopian zones was also displayed in Fig. 2. The spatial patterns of the adjusted HIV odds ratio across Ethiopian zones showed significant variations in HIV prevalence. After adjusting for confounding variables, the results indicated that there were substantially significant spatial variations between location and HIV prevalence since the *p* value to evaluate the global spatial effect of HIV was 0.000 (Table 3). The optimal span size increased from 15% (in crude) to 50% of the data following the adjustment for variables, and the global residual deviance value was decreased (Table 3). The range of odds ratios in the study areas was also substantially narrowed after adjustment (AOR, range: 0.05–4.31), compared to an unadjusted (COR, range: 0–7.44). Moreover, the first quartile, the mean, and the third quartile of the variables effect prediction for both crude and adjusted covariates, showing that the adjusted one is contracted.

In general, spatial adjustment for covariates decreased the size with geographical zones of higher odds ratio compared to the counter parts of the crude. In Central Ethiopia, the odds ratio for HIV was significantly reduced after adjusting for covariates. Additionally, it has slightly decreased in the North Eastern regions of the nation and the Gambella regional zones. The lower odds ratio of HIV was also slightly shifted from the eastern to southern parts of Somalia region.

The *p* value of the effects of the prediction of variables and adjusting for individual covariates are presented in Table 4. The *p* values for each variable under the GAMs model are significant, which indicates that each variable has a potential contribution to the prevalence of HIV risk. Therefore, using these prediction points and the odds mapping smoothed spatial effect estimates from individual-level data were successful.

**Table 4:** Adjusted for each variable prediction effect and statistic.

	Deviance	AIC	*p* value	Variables effect predictions		
				Minimum	Mean	Maximum
Place of residence	3813.96	3833.00	0.000	− 3.43	− 0.35	1.57
Current marital status	4005.82	4027.00	0.000	− 3.38	− 0.32	1.73
Had any STI in the last 12 month	4021.03	4041.15	0.000	3.49	− 0.34	1.70
Educational level	3925.09	3949.14	0.000	− 3.16	− 0.31	1.61
Age at first sex	4030.84	4054.95	0.000	− 3.45	− 0.34	1.73
Ever been tested for HIV	3977.53	3997.65	0.000	− 2.97	− 0.27	1.75
Ever heard of AIDS	4026.13	4046.22	0.000	− 3.30	− 0.31	1.81
Ever used contraceptive method	4018.90	4039.01	0.000	− 3.30	− 0.31	1.78

**Figure 3:**
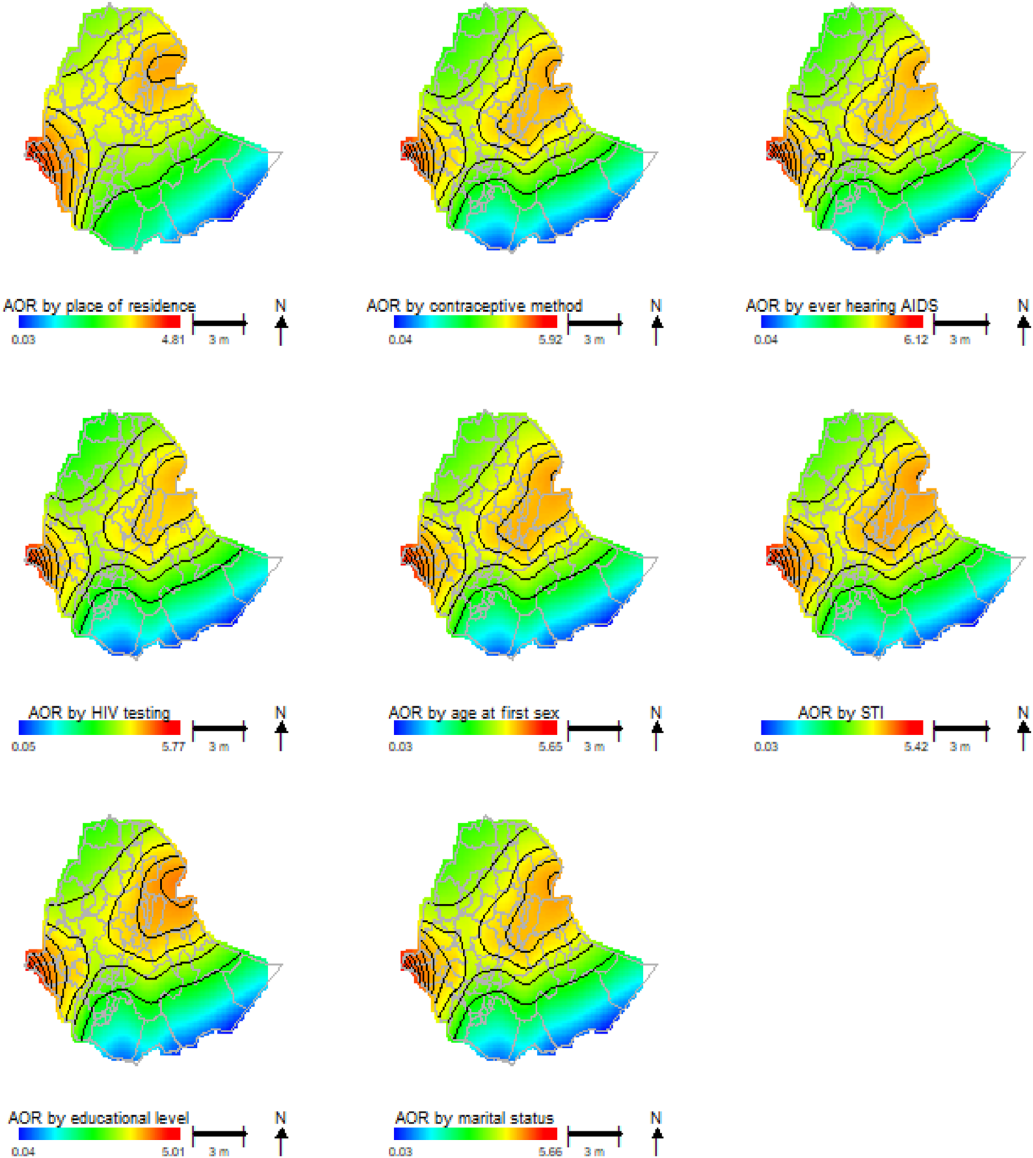
Contributions of potential risk factors to the spatial distributions of HIV prevalence in terms of Odds Ratio. The bold black contour lines indicate areas where the upper and lower bands exclude one.

This study examined the potential contributions of each sociodemographic predictors on HIV prevalence. Figure 3 shows the potential contributions of predictors such as place of residence, current marital status, educational level, had a sexual transmitted disease for the last 12 months, age at first sex, had ever been tested for HIV/AIDS, having ever heard HIV and had ever used contraceptive method to geographical patterns of HIV odds ratio associated with the geo locations (longitude, latitude). The highest odds ratios of HIV for each predictor variable were smaller than the crude odds ratio and larger than the summary adjusted for covariates HIV odds ratio. In addition, the spatial disparities of the HIV odds ratio of each variable have a similar pattern as the adjusted covariates map in Fig. 2. Also, the HIV odds ratio of each potentially contributing variable was slightly different from each other in spatial distributions. The spatial patterns of the HIV odds ratio adjusting for place of residence were significantly reduced from the crude odds ratio. The highest odds ratio of HIV was significantly reduced in the Eastern, Central and Western parts of the country for adjusting the place of residence.

## Discussion

This study used the 2016 EDHS data adopted with GAMs to smooth the two dimensional spatial effects. The spatial components were the bivariate location (longitude, latitude) associated with the respondents and the zonal shape files to produce zonal-level results. The result found spatial disparities of HIV prevalence based on the individual level geo locations and adjusted for covariates. Variations of geo locations and magnitudes of HIV odds ratio were observed in both crude and adjusted spatial effects. Spatial variations among HIV prevalence revealed a significant shift in spatial patterns of HIV prevalence.

For crude analysis, the odds of HIV infection were significantly higher in western, central, and eastern parts of the country. It is a similar spatial pattern for covariate adjustments by reducing the odds ratio from the crude analysis. After adjusting for covariates, either for all variables (Fig. 2) or each individual variable (Fig. 3), the odds ratio of HIV prevalence was reduced. In addition to reducing the odds, we found that significant zones with the lowest odds ratio of HIV (low HIV risk zones) were shifted from the Degahabur zone to the Shebelle and Liben zones in the eastern parts of the country.

The results of adjusting for individual covariates for significant factors displayed substantial spatial variations across zones. As of the adjusted for covariates HIV odds ratio mapping, the individual adjustment variable had significant spatial variations across the Ethiopian zones. Our findings agree with similar studies^17,33,39^. We found that the higher or lower HIV prevalence adjusted for place of residence had significant spatial disparities, which reduced the odds ratio of crude analysis.

The findings of the SSA study^23^ revealed that HIV prevalence maps produced spatial disparities in the epidemic within a nation and localized areas where both the burden and causes of the epidemic are concentrated. According to research by^40^, recently pregnant women in a rural area of Kenya reported spatial variability in the reporting of externalized HIV stigma. The studies conducted in South Africa examined spatial differences in prevalence in South African provinces, and their findings indicate spatial variability in HIV prevalence^33^. In addition, their study looked at how the GAM model could be used to smooth out the effects of geographical location by increasing and decreasing the chances of HIV in statistically significant areas. Our investigation supported these findings, showing that there were geographic disparities in the highest and lowest odds of HIV prevalence in Ethiopian zones.

There have been geographical analysis studies connected to community and regional levels in Ethiopia, even though we are aware of no studies using spatial disparities of HIV prevalence throughout the nation's geographic zones. According to the research of^7^, there are significant spatial variations in the national HIV risk at the subnational and local levels. Furthermore, their study found an association between demographic factors and the spatial distribution of HIV, with the Gambella region having the highest prevalence of the disease. Agnuak and Nuer zones of the Gambella region were found to have the highest HIV prevalence according to our study, which was consistent with their findings. In Ethiopia, trends and spatial distributions of HIV prevalence have been studied^7^. Their findings, which are consistent with ours, show that Addis Ababa and the neighboring regions of the Afar, Tigray, and Amhara regional states, as well as central Oromia, consistently have high clusters of HIV cases.

Our study had some quality in assessing the geo-mapping of the HIV odds ratio for unadjusted and adjusted covariates using a recently developed *MapGAM* package. We use GAMs to find geographical disparities in HIV risk at the zonal level. GAMs allowed us to identify geographical variations and support local regression and spatial splines with nonparametric smoothing effects. We still have some limitations despite our strengths. Due to our study being limited to the EDHS data, some medical prevention intervention factors, such as those related to anti-retroviral (ART) therapy, were left out^26^.

## Conclusion

HIV prevalence is spatially varied in Ethiopian zones. Some zones were highly affected by HIV risk, and others were looking safe. The study displayed significant areas of high and low HIV prevalence zones. In particular, the western, part of eastern to the north and central parts of Ethiopian zones were affected by HIV, while part of Eastern to the southern Ethiopia had low HIV prevalence. People who were living in the rural areas, had a secondary educational level, had had any STIs for the last 12 months prior to the survey, and used contraceptive methods were more likely to be affected by HIV disease than their respective counterparts. Health policy makers should intervene in high-HIV prevalence zones to achieve the strategies designed to eliminate HIV/AIDS endemics by 2030.

### Supplementary Information

Supplementary Information.

## Data Availability

The 2016 EDHS HIV data was accessed from the DHS program website https://www.dhsprogram.com and also, the enumeration area and zones shapefiles were accessed from https://www.dhsprogram.com and https://africaopendata.orgg websites respectively, after a request for registration.
