# Supplementary material for: Mapping the spatial disparities of HIV prevalence in Ethiopian zones using the generalized additive model
Source: Sci Rep. 2024 Mar 14;14:6215. doi: 10.1038/s41598-024-55850-8 (PMC10940621; doi:10.1038/s41598-024-55850-8)
Supplement: Supplementary file 1 — Supplementary Information. [file 41598_2024_55850_MOESM1_ESM.docx]

The general R coding for the analysis specifically for the COR, AOR and mapping is given as follows

**library(MapGAM)**

**library(rgdal)**

**library(rgeos)**

**library (sf)**

**library(stars)**

**library(sp)**

**library(raster)**

**library(tidyverse)**

**library(maptools)**

**HIV_data<-read.csv("D:/Further Research Areas/geoGAM/HIV_GAM.csv")**

**Ethio=readShapePoly("D:/for PhD studies/PhD Desertation/Data Description/ETH_adm/ETH_adm2.shp", proj4string=CRS(as.character(NA)), verbose=F, repair=FALSE, force_ring=T, delete_null_obj=T, retrieve_ABS_null=T)**

**#For trimed data**

**library("PBSmapping")**

**trimdata(HIV_data, E)**

**gamgrid <- predgrid(HIV_data, map = Ethio)**

**obj <- list(grid=data.frame(HIV_data$ddlong,HIV_data$ddlat),fit=HIV_data$HIV)**

**colormap(fit_gam01, Ethio, legend.name = "HIV")**

**# plot the grid points**

**plot(gamgrid$ddlong, gamgrid$ddlat, cex=0.1, col="red")**

**# and the data locations**

**points(Ethio$ddlong,Ethio$ddlat)**

**fit_gam01 <- modgam(data = HIV_data, rgrid = gamgrid, m = "crude", sp = 0.15, verbose = T)**

**fit_gam02 <- modgam(HIV ~ lo(ddlong, ddlat)+factor(Resid)+**

**factor(Edu)+factor(Mstatus)+factor(STI)+factor(AgeFsex)+**

**factor(HIVTest)+factor(EHADIS)+factor(Contraceptive),**

**data = HIV_data, rgrid = gamgrid,**

**family = binomial(), sp = 0.5, verbose = T)**

**#par(mfrow=c(1,2))**

**##To map the COR and AOR for each zones**

**colormap(fit_gam01, exp =T, Ethio, contours = "response", border.gray = 0.7,**

**legend.name = "Crude Odds Ratio ", legend.cex = 0.85)**

**colormap(fit_gam02, exp = T, Ethio, contours = "response", border.gray = 0.7,**

**legend.name = "Adjusted Odds Ratio", legend.cex = 0.85)**

**#The spatially ajdusted Odss Ratio using individual covariates**

**fit_region = modgam(HIV ~ lo(ddlong, ddlat)+factor(region),**

**data = HIV_data, rgrid = gamgrid, sp = NULL)**

**fit_Resid = modgam(HIV ~ lo(ddlong, ddlat)+factor(Resid),**

**data = HIV_data, rgrid = gamgrid, sp = 0.5)**

**fit_Edu = modgam(HIV~lo(ddlong,ddlat)+factor(Edu),**

**data = HIV_data, rgrid = gamgrid, sp = 0.5)**

**fit_MS = modgam(HIV~lo(ddlong,ddlat)+factor(Mstatus),**

**data = HIV_data, rgrid = gamgrid, sp = 0.5)**

**fit_STI = modgam(HIV~lo(ddlong,ddlat)+factor(STI),**

**data = HIV_data, rgrid = gamgrid, sp = 0.5)**

**fit_AgeF = modgam(HIV~lo(ddlong,ddlat)+factor(AgeFsex),**

**data = HIV_data, rgrid = gamgrid, sp = 0.5)**

**fit_HIVT = modgam(HIV~lo(ddlong,ddlat)+factor(HIVTest),**

**data = HIV_data, rgrid = gamgrid, sp = 0.5)**

**fit_EADIS = modgam(HIV~lo(ddlong,ddlat)+factor(EHADIS),**

**data = HIV_data, rgrid = gamgrid, sp = 0.5)**

**fit_cont = modgam(HIV~lo(ddlong,ddlat)+factor(Contraceptive),**

**data = HIV_data, rgrid = gamgrid, sp = 0.5)**

**#Maping spatially covariate adjusted indvidual variables**

**colormap(fit_Resid, exp =T, Ethio, contours = "response", border.gray = 0.7,**

**legend.name = "AOR by place of residence", legend.cex = 0.85)**

**colormap(fit_cont, exp =T, Ethio, contours = "response", border.gray = 0.7,**

**legend.name = "AOR by contraceptive method", legend.cex = 0.85)**

**colormap(fit_EADIS, exp =T, Ethio, contours = "response", border.gray = 0.7,**

**legend.name = "AOR by ever hearing AIDS", legend.cex = 0.85)**

**colormap(fit_HIVT, exp =T, Ethio, contours = "response", border.gray = 0.7,**

**legend.name = "AOR by HIV testing", legend.cex = 0.85)**

**colormap(fit_AgeF, exp =T, Ethio, contours = "response",border.gray = 0.7,**

**legend.name = "AOR by age at first sex", legend.cex = 0.85)**

**colormap(fit_STI, exp =T, Ethio, contours = "response",border.gray = 0.7,**

**legend.name = "AOR by STI", legend.cex = 0.85)**

**colormap(fit_Edu, exp =T, Ethio, contours = "response",border.gray = 0.7,**

**legend.name = "AOR by educational level", legend.cex = 0.85)**

**colormap(fit_MS, exp =T, Ethio, contours = "response",border.gray = 0.7,**

**legend.name = "AOR by marital status", legend.cex = 0.85)**

**#colormap(fit_region, exp =T, Ethio, contours = "response",border.gray = 0.7,**

**legend.name = "AOR by region", legend.cex = 0.85)**
